# Healthy Aging Eyes & Ears: Sensory Health Needs and Barriers for Adults Aging with HIV

**DOI:** 10.1093/geroni/igaf122.4383

**Published:** 2025-12-31

**Authors:** Alison Abraham, Erin Burk-Leaver, Hannah Kisselburgh, Haley Sanner

**Affiliations:** University of Colorado--Denver, Aurora, Colorado, United States; Colorado Health Network, Denver, Colorado, United States; Colorado School of Public Health, Aurora, Colorado, United States; Colorado Health Network, Denver, Colorado, United States

## Abstract

Sensory loss is common among aging adults and has been linked to poor health outcomes, including depression, frailty, cognitive decline, falls, and poor quality of life. Vision and hearing loss are more prevalent among adults aging with HIV (AAWH) and are associated with decreased healthcare engagement. This qualitative study used a community-based participatory research approach to explore sensory health experiences, needs, and program preferences of AAWH receiving services at Colorado Health Network, a statewide AIDS Service Organization in Denver. Following purposive sampling, we conducted and audio-recorded ten individual interviews and two focus groups with 23 participants (ages 51-73), who shared their lived experiences with sensory loss and perspectives on sensory care. Audio recordings were transcribed, and data were analyzed through deductive and inductive thematic analysis and team-based triangulation. Six themes were identified: interplay of HIV and aging in sensory decline perceptions; sensory aid stigma and self-perceptions of accelerated aging; navigating affordability of sensory aids; creative behavioral, environmental, and technological adaptations to sensory loss; long-term survivorship as a driver of self-management of care and self-advocacy; integrating ideal sensory care for AAWH. Using the Social Ecological Model, we identified facilitators and barriers to sensory care at the individual, interpersonal/provider, organizational/community, and system levels. Facilitators included motivation to maintain independence, peer modeling, community-based benefits, and care navigation. Barriers included discomfort with assistive devices, stigma, provider bias, limited awareness of HIV-specific health needs, and gaps in insurance coverage. Findings offer guidance for designing community-based sensory health programs tailored to the lived experience of AAWH.

